# Data on stable isotopic composition of δ^18^O and δ^2^H in precipitation in the Varaždin area, NW Croatia

**DOI:** 10.1016/j.dib.2020.106573

**Published:** 2020-11-23

**Authors:** Igor Karlović, Tamara Marković

**Affiliations:** Croatian Geological Survey, Milana Sachsa 2, Zagreb 10000, Croatia

**Keywords:** Water stable isotopes, Oxygen and hydrogen, D-excess, Precipitation, Local meteoric water line, Varaždin area

## Abstract

The dataset in this article consists of one year of monthly observations of water stable isotopes in precipitation (July 2019 - May 2020). The samples were collected by rain gauge installed in the Hrašćica village in the Varaždin area, NW Croatia. The data presented in the article are raw isotope data of precipitation supplemented by calculated d-excess values, and local meteoric water lines (LMWL). The measured data is an extension to previous two year investigations (June 2017 – June 2019), which was published in the research article “Application of Stable Water Isotopes to Improve Conceptual Model of Alluvial Aquifer in the Varaždin Area” [1]. Local meteoric water lines (LMWL) are calculated for the entire period for which isotope analyses exist (June 2017 - May 2020). Presented data can be used as a background for investigation of precipitation, groundwater and surface water origin and their interrelationships.

## Specifications Table

**Table:** 

Subject	Atmospheric Science
Specific subject area	isotope hydrology
Type of data	Table Graph Figure
How data were acquired	Monthly composite precipitation samples collected in the field, ratios of water stable isotopes measured using Picarro L2130-i Isotope analyser. Monthly amount of precipitation obtained from Croatian Meteorological and Hydrological Service for the synoptic meteorological station Varaždin.
Data format	Raw analysed
Parameters for data collection	Monthly composite precipitation collected by a special designed rain gauge for collection of precipitation samples for stable isotope analysis. Prior to isotope analyses, samples were filtered through 0.45 μm sterile syringe filters (Chromafil Xtra PET-45/25) to remove impurities.
Description of data collection	From July 2019 to May 2020, fieldwork was conducted to collect 10 precipitation samples for water stable isotope analysis. Ratios of water stable isotopes expressed as δ^18^O and δ^2^H were measured with CRDS (Cavity Ring-Down Spectroscopy) technology in the Hydrochemical Laboratory of the Croatian Geological Survey. The deuterium excess (d-excess) was calculated for each sample using the equation: *d* = δ^2^H – (8 x δ^18^Ο). The local meteoric water line (LMWL) was calculated using Local Meteoric Water Line Freeware [2]. Input data for construction of LMWL consist of 34 analysed samples (10 samples from this investigation and 24 samples from previous investigation [1]).
Data source location	Rain gauge located in the Hrašćica village, near the town Varaždin - Varaždin County, NW Croatia (46.32516°N, 16.29311°E, 177 m a.s.l).
Data accessibility	With the article
Related research article	T. Marković, I. Karlović, I., M. Perčec Tadić, O. Larva, Application of Stable Water Isotopes to Improve Conceptual Model of Alluvial Aquifer in the Varaždin Area. Water, Special Issue Use of Water Stable Isotopes in Hydrological Process, 12, 379 (2020) https://doi.org/10.3390/w12020379[1]

## Value of the Data

• Presented data provide an insight into seasonality of stable isotope ratios in precipitation, which is highly important component of the hydrological cycle, and associated local meteoric water lines serve as background for determination of the groundwater and/or surface water origin. In addition, the relationship between ratios δ^18^O, δ^2^H, and d-excess enables the determination of air masses from which the rainfall originates [1].
• Researchers working on hydrology/hydrogeology related problems can use the data for identifying the origin of precipitation, groundwater and surface water and determination of their interrelationships.
• The data can be used as input for regional studies, e.g. investigation of isotope effects (seasonal, altitude, continental effect) in modern precipitation [3].

## 1 Data Description

**Fig. 1:**
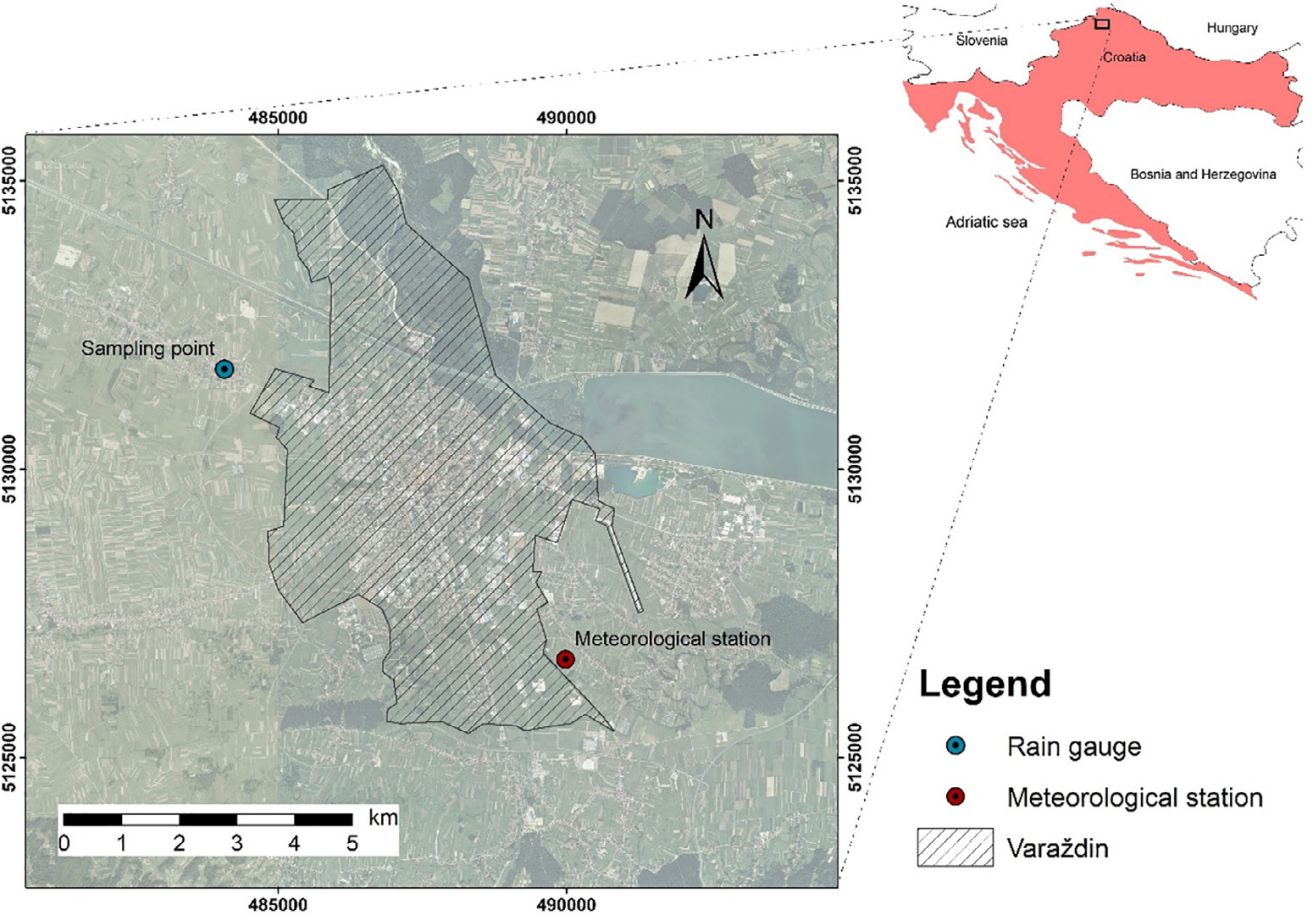
Map of the Varaždin area with location of the sampling point in the village Hrašćica and meteorological station Varaždin.

The data presented include water stable isotope ratios (δ^18^O and δ^2^H) of monthly composite precipitation collected by rain gauge in the village Hrašćica, near the town Varaždin, NW Croatia (46.32516°N, 16.29311°E, 177 m a.s.l) in the period from July 2019 to May 2020. The location of the sampling point is shown in Fig. 1.

**Fig. 2:**
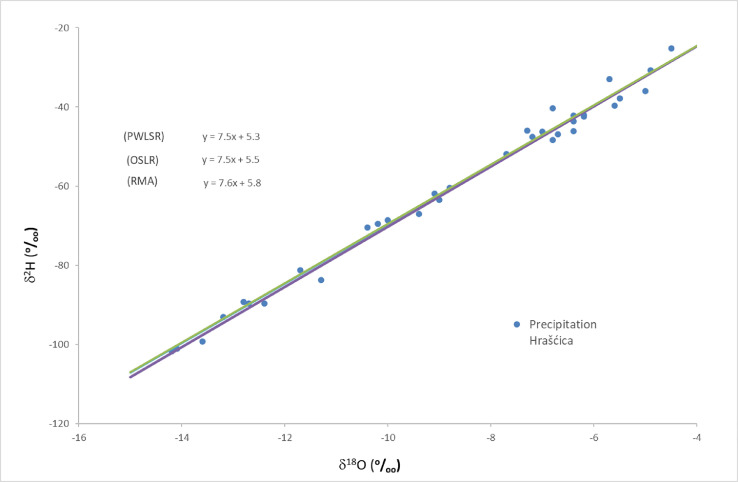
Dual isotope diagram showing monthly stable isotope ratios measured in 34 precipitation samples from Hrašćica, with calculated local meteoric water lines for the period June 2017 - May 2020.

Table 1 shows stable isotope ratios δ^18^O and δ^2^H measured values with associated d-excess values from samples collected by rain gauge in Hrašćica and monthly amount of precipitation measured at the meteorological station Varaždin. The isotope data encompasses 10 individual precipitation stable isotope analyses. During the sampling period, February 2020, the inlet of the rain gauge was clogged and the collected precipitation amount was insufficient for isotope analysis. A total of 34 analyses were used for calculation of local meteoric water line (LMWL), 10 measurements from this sampling period (July 2019 – May 2020) and 24 measurements from previous sampling period (June 2017 – June 2019). Measured δ^18^O and δ^2^H are plotted on dual isotope diagram, together with calculated local meteoric water lines and their equations (Fig. 2).

**Table 1:** Measured stable isotope ratios δ^18^O and δ^2^H values in 10 precipitation samples from Hrašćica, with associated d-excess values, and monthly precipitation measured at the meteorological station Varaždin.

Month/Year	δ^18^Ο (^o^/_oo_)	δ^2^H (^o^/_oo_)	d-excess (^o^/_oo_)	Precipitation amount (mm)
VII/2019	−6.2	−42.1	7.5	142.5
VIII/2019	−5.7	−33.0	12.6	93.2
IX/2019	−6.8	−40.4	14.0	71.6
X/2019	−7.3	−46.0	12.4	32.7
XI/2019	−10.0	−68.6	11.4	144.4
XII/2019	−13.2	−93.1	12.5	120.0
I/2020	−10.2	−69.5	12.1	29.0
II/2020*	–	–	–	25.9
III/2020	−10.4	−70.5	12.7	41.9
IV/2020	−6.7	−46.9	6.7	23.2
V/2020	−6.4	−46.1	5.1	49.9

⁎ the amount of collected precipitation was insufficient for isotope analysis.

The calculated LMWL for the period from June 2017 to May 2020 is:

$$\text{OLSR}\;\delta^{2}H=\left(7.5\pm 0.1\right)\;\delta^{18}O+\left(5.5\pm 1.2\right),\;n=34$$

$$\text{RMA}\;\delta^{2}H=\left(7.6\pm 0.1\right)\;\delta^{18}O+\left(5.8\pm 1.2\right),\;n=34$$

$$\text{PWLSR}\;\delta^{2}H=\left(7.5\pm 0.1\right)\;\delta^{18}O+\left(5.3\pm 1.2\right),\;n=34.$$

Supplementary data files submitted with the article:

**Rain gauge.zip** – ESRI shapefile with exact location of the sampling point in Hrašćica. Coordinate reference system is the official coordinate system of the Republic of Croatia (HTRS96/TM).

**Meteo station.zip** – ESRI shapefile with exact location of the meteorological station Varaždin. Coordinate reference system is the official coordinate system of the Republic of Croatia (HTRS96/TM).

**Stable_isotopes_Hrascica.xlsx** – table with values of measured data, associated d-excess values and monthly precipitation (Table 1), dual isotope diagram (Fig. 2), and the calculation of LMWL shown in Fig. 2.

**Map.jpg** – Map of the Varaždin area with location of the sampling point in Hrašćica and meteorological station Varaždin (Fig. 1).

**Dual_isotope_diagram.pdf** – graph representing monthly stable isotope ratios in 34 precipitation samples from Hrašćica, with local meteoric water lines (Fig. 2).

## 2 Experimental Design, Materials and Methods

Monthly composite precipitation samples (*N* = 10) were collected using by a special designed rain gauge for collection of precipitation samples for stable isotope analysis, which was installed in the village Hrašćica (46.32516°N, 16.29311°E, 177 m a.s.l), near the town Varaždin - Varaždin County, NW Croatia. Field sampling was conducted on a monthly basis in duration of one year, from July 2019 to May 2020. The special designed rain gauge (which is used by IAEA) collected precipitation into 3 L HDPE container which is installed in the gauge. The last day of the month, composite sample was poured into 1000 mL or in smaller or bigger HDPE plastic bottles with a tight-fitting cap (depending on how much sample was collected during the month), and preserved at 4 °C in the portable refrigerator until analysis. To remove impurities, all samples were filtered through 0.45 μm sterile syringe filters (Chromafil Xtra PET-45/25) before isotope analyses. Analyses were done upon arrival of the sample into the laboratory.

Stable isotope ratios in precipitation were analysed in the Hydrochemical Laboratory of the Croatian Geological Survey, using Cavity Ring-Down Spectrometer Picarro L2130-i Isotope analyser [4], with a precision of ± 0.3 ‰ for δ^18^Ο and ± 1 ‰ for δ^2^H. All measurements were checked with Picarro's standards (Depleted −29.6 ± 0.2 δ^18^Ο; −235 ± 1.8 δ^2^H; Mid −20.6 ± 0.2 δ^18^Ο; −159 ± 1.3 δ^2^H; Zero 0.3 ± 0.2 δ^18^Ο; 1.8 ± 0.9 δ^2^H), which were checked periodically against the International Atomic Energy Agency (IAEA) standards: Vienna Standard Mean Ocean Water 2 (VSMOW2) and Standard Light Antarctic Precipitation 2 (SLAP2). To achieve accurate measurements, elimination of “bad” samples or injections based on H_2_O concentrations due to syringe or septa underperformance, between-sample ‘’memory-effect’’, determination δ amount dependence on H_2_O amount, determination and application of residual memory carryover correction, correction for linear or non-linear instrumental drift, and normalization of all data to the VSMOW/SLAP scales, IAEA guidance was used [5]. The measured values are expressed in standard δ-notation (‰) [6, 7]. The deuterium excess (d-excess) was calculated for each sample using the equation: *d* = δ^2^H – (8 × δ^18^Ο) [8].

The local meteoric water line (LMWL) has been calculated using Local Meteoric Water Line Freeware [2]. Three types of linear regression analysis were performed, two of which are recommended by the IAEA [9]: ordinary least squares regression (OLSR) and reduced major axis (RMA) regression. The third one takes into account the amount of precipitation, using precipitation weighted least squares regression (PWLSR) [2]. The monthly amount of precipitation needed for PWLSR analysis was provided by Croatian Meteorological and Hydrological Service (Table 1) for the synoptic meteorological station Varaždin (46.28240°N, 16.36385°E, 167 m a.s.l) (Fig. 1). The station is part of the Croatian national meteorological network and obtained data is publicly available at [10].

## Declaration of Competing Interest

The authors declare that they have no known competing financial interests or personal relationships which have, or could be perceived to have, influenced the work reported in this article.

## Appendix Supplementary materials

Image, application 1
