# Supplementary figures and images for: Data on stable isotopic composition of δ18O and δ2H in precipitation in the Varaždin area, NW Croatia
Source: Data Brief. 2020 Nov 23;33:106573. doi: 10.1016/j.dib.2020.106573 (PMC7708787; doi:10.1016/j.dib.2020.106573)

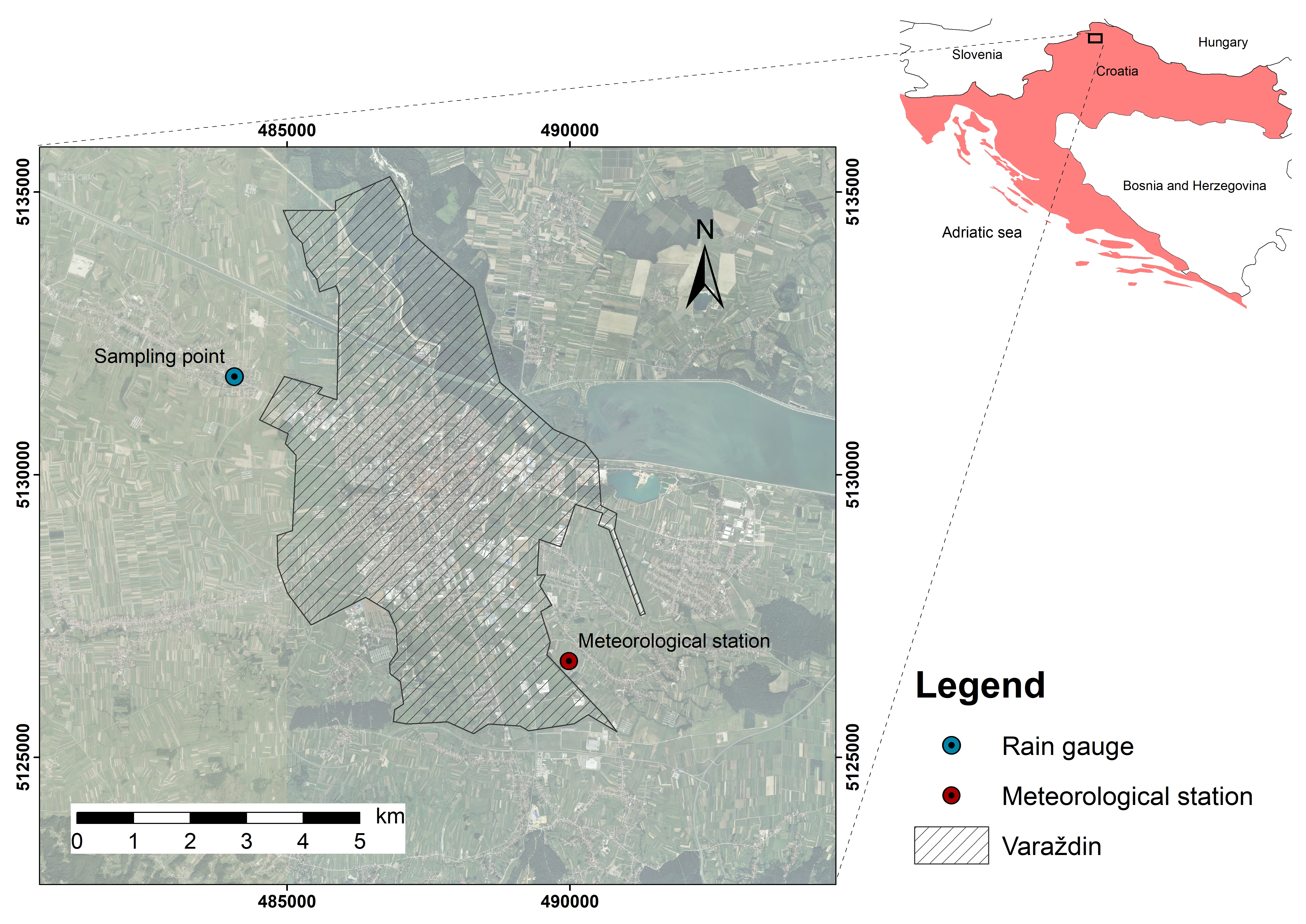

Supplement: Supplementary file 1 [file mmc1.zip › Supplementary material/Map.jpg]

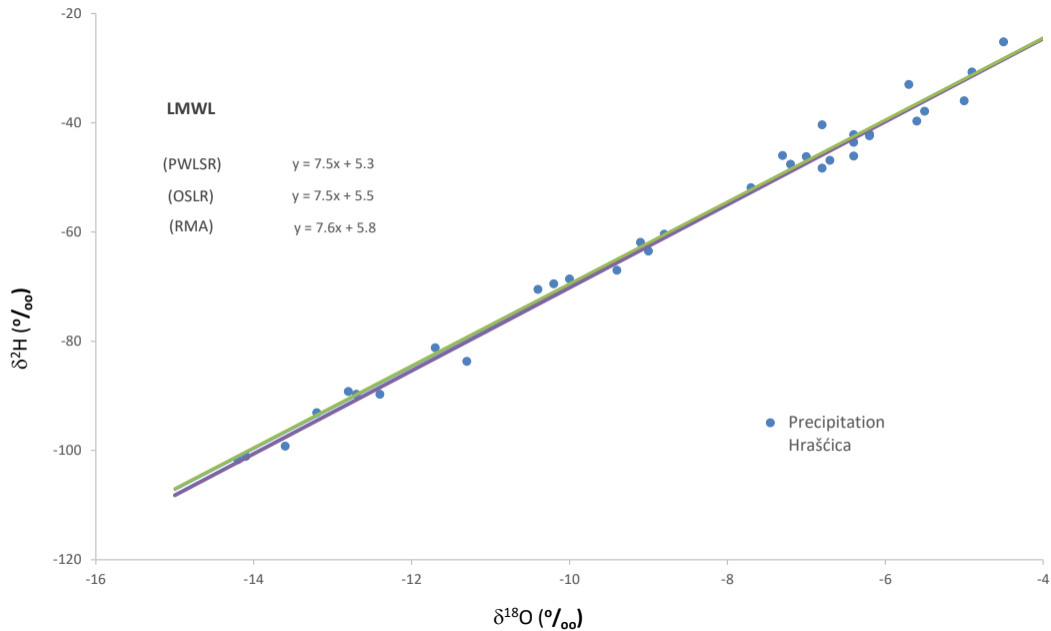

Supplement: Supplementary file 1 [file mmc1.zip › Supplementary material/Dual_isotope_diagram.pdf]
